# Defusing the Neurosurgery Arms Race: A Blueprint for Quality-Focused Residency Selection

**DOI:** 10.1227/neu.0000000000003904

**Published:** 2026-01-09

**Authors:** Austin M. Hilvert, Sameer Sundrani, Clayton R. Baker, Campbell Liles, Lola B. Chambless

**Affiliations:** Department of Neurological Surgery, Vanderbilt University Medical Center, Nashville, Tennessee, USA

**Keywords:** Residency match, Research, Education, Applications, Applicants, Medical student, Academic, Arms race

## Abstract

Publication expectations in neurosurgery residency selection have intensified as traditional objective metrics have receded, creating a zero-sum “arms race” that prioritizes volume over impact. Drawing on literature from medical education, psychology, and game theory, this article examines how raw publication counts function as imperfect signals of merit. They disproportionately disadvantage applicants without access to robust research infrastructure, compress timelines, incentivize low-impact output, and contribute to anxiety and burnout. We propose a framework to realign incentives toward rigor, equity, and clinical relevance. Key recommendations include adopting quality-weighted scoring systems for scholarly work (e.g., the evolving Arms Race Control Score), capping the number of research items applicants may report, and requiring concise statements that define an applicant's scholarly trajectory. Additional measures include formally recognizing impactful nontraditional contributions (e.g., quality improvement projects, open-source tools, patents, device innovations), piloting objective assessments relevant to neurosurgery (such as simulation-based surgical skills and standardized subinternship milestones), and committing to longitudinal research evaluating whether pre-residency publications predict training and career outcomes. Implemented collectively and supported by shared program director guidelines, these reforms could shift focus from publication counts to meaningful scholarship, preserve opportunities for late-deciding applicants and those at less-resourced institutions, and strengthen the scientific foundation of residency selection. Neurosurgery is uniquely positioned to lead this transition and set a precedent for the broader medical community.

**ABBREVIATION:**
ARCS: Arms Race Control Score.

In recent years, medical students—particularly those applying to highly competitive specialties such as neurosurgery—have significantly increased their research output.^1-3^ The volume of publications featuring medical student authors has surged over the past decade, paralleled by a rise in the number of coauthors per neurosurgical paper.^1-4^ Some neurosurgeon leaders have expressed concern that this phenomenon may be driven more by the pressures of residency selection than by genuine scientific curiosity.^5-8^

One major accelerant of this dynamic has been the reduction of objective performance metrics in residency applications. Over the past 2 decades, many medical schools have eliminated grades, class ranks, and honors such as Alpha Omega Alpha.^9-13^ The transition of United States Medical Licensing Examination Step 1 to pass/fail further removed a standardized measure of academic comparison. Proponents of these changes often cite 2 primary motivations: promoting student well-being by reducing anxiety-inducing metrics and addressing concerns that certain groups may be disadvantaged by implicit bias.^9,10^ In the absence of such standardized benchmarks, residency programs have placed greater emphasis on other components of the application—most notably, research.^14^ In the absence of objective scoring systems, publication count may function as a proxy for qualities such as academic ability and motivation, despite its limitations as a measure of these traits.^14,15^

The pressure to publish—once reserved for established academics—has filtered down to medical students, embedding a “publish or perish” mindset. Faculty incentives further drive students—often unpaid—to generate “high-yield” research. While a strong publication record can reflect diligence and potential—especially for aspiring surgeon-scientists—the emphasis on quantity carries significant drawbacks. Emerging technological tools have made it easier to produce superficial content, and students at institutions with fewer research resources are placed at a disadvantage, undermining equity in the selection process.

Neurosurgery, with its long-standing emphasis on academic output, is well-positioned to collaborate with broader Graduate Medical Eduation leadership in rethinking how residency applicants are evaluated. The field's commitment to research provides an opportunity to realign the metrics of academic success away from sheer volume and toward quality, authenticity, and impact. This is essential—not only to ensure fairness in selection but also to promote a neurosurgical workforce driven by purpose rather than performance metrics. Through psychological and economic lenses—such as signaling theory, incentive design, and game theory—we examine the roots of the current publication arms race and propose structural reforms to realign incentives toward meaningful scholarship.

## ESCALATING OUTPUT, DIMINISHING RETURNS

The residency match operates as a zero-sum game: the number of available residency positions is fixed, meaning that an improvement in one applicant's relative standing necessitates a decreased probability of success for others. In such a system, outcomes are not determined by absolute merit but by relative performance.^16^ When research productivity emerges as a key metric, it creates an asymmetric cost-benefit dynamic: those who publish more may marginally improve their chances, but this escalation devalues the publication efforts of others, prompting a feedback loop of ever-increasing output. This reflects what game theorists describe as a “positional arms race,” where the utility of a signal (e.g., publication count) depends entirely on its ability to exceed the norm, creating incentives for overproduction despite diminishing returns.^16-21^

In this context, research output shifts from being an authentic measure of scholarly aptitude to a strategic tool used to maintain relative competitiveness. Game theory describes this inefficiency as individual rationality producing collective suboptimality.^19-21^ Across specialties, applicant research volume has surged so dramatically that today's unmatched applicants often have more research credits than the average matched applicant did 10 years ago.^1-4,22,23^

Neurosurgery applicants have followed—and in some cases led—this trend. A recent analysis of 2017-2021 neurosurgery matriculants found a mean of ∼5.6 peer-reviewed manuscripts (median of 3) before residency, with a significant year-by-year increase over that period.^6,24,25^ This continues a decades-long pattern: since the 1980s, each generation of neurosurgery trainees has published more before board certification than the last,^26^ reflecting accelerating early-career expectations.

Much of this burgeoning output may have limited scientific impact. One study found that most medical student-authored papers are case reports or reviews, and 59% lack even one citation.^27^ This raises the question of whether the current system is generating “research pollution”—an oversaturation of low-impact publications. Underreporting of negative results and statistical errors further distort the evidence base.^28,29^ As a result, even applicants with lengthy publication records may present work of widely variable quality and scientific value—highlighting the need for more objective and equitable evaluation methods.

## STRUCTURAL BARRIERS AND COMPRESSED TIMELINES

Importantly, the burden of this arms race is not evenly distributed. Students from institutions without strong research infrastructure, home neurosurgery programs, or mentorship are inherently disadvantaged.^30-32^ One study found that neurosurgery interns from top 20 medical schools had significantly more publications than those from lower-ranked programs.^6^ Although this may reflect real institutional strengths, a system that rewards research quantity amplifies disparities and risks selecting for privilege rather than promise.

Crucially, many students do not choose the most prestigious medical school they are accepted to. Financial constraints, proximity to family, and guidance from mentors often lead them to select more affordable or local programs. These rational choices may inadvertently limit future opportunities in ways that are neither obvious nor fair. The assumption that students at the highest-ranked schools are inherently the “best” often ignores the advantages that paved their way as well as the rational reasons other students may have opted for less prestigious schools.

In addition, students who choose neurosurgery later in medical school have less time to build a competitive academic profile. This may punish those entering medical school with a broad range of interests and reward those choosing a highly specialized career before they have had thorough exposure to a variety of options. As a result, many “latecomers” feel compelled to take research years, extending their training and increasing financial strain. The current system rewards those with early exposure and institutional resources while reinforcing structural barriers to entry.

## MISALIGNED INCENTIVES

With the United States Medical Licensing Examination Step 1 now pass/fail—and similar proposals under discussion for Step 2—the landscape for applicant differentiation has narrowed considerably.^14^ Students are left with a limited set of domains through which to distinguish themselves: academic grades (subject to inflation), extracurricular engagement, letters of recommendation, and scholarly output. In the absence of more granular metrics, publication volume is often interpreted as a proxy for academic rigor, motivation, and potential. Program Director surveys consistently rank “research experience” as a significant criterion for interview selection in competitive specialties such as neurosurgery.^33^

Thus, students respond rationally to the incentive: a higher number of publications is perceived as a positive signal of academic capability, thereby enhancing their competitiveness as applicants. However, signaling theory cautions that such signals are often imperfect and potentially inefficient—they may be costly to produce and may not accurately reflect the qualities they are intended to convey.^15,34^

As Douglas Altman^35^ warned, career pressures can lead to poorly designed, low-value research. When programs reward raw publication counts, students may prioritize quick-to-publish projects or fragment work into smaller outputs. In some cases, this incentivizes superficial or even dishonest research.^36-38^ Emphasizing quantity comes at the expense of deeper, more meaningful scholarship.

Healthy competition can motivate productivity, but a hypercompetitive, zero-sum environment undermines motivation quality. Psychologically, students may shift from intrinsic motivation (“I'm curious and want to solve a problem”) to extrinsic motivation (“I need another publication to match into neurosurgery”).^39^ A 2021 survey of medical students in neurosurgery research found their top motivators were genuine interest and competitiveness; 66% reported anxiety about publication volume and time to do research.^5^ Instead of joy in discovery, many feel pressured to produce. Over time, this environment risks fueling burnout and a transactional approach to research.

## PROPOSALS FOR ADDRESSING THESE ISSUES

To address these challenges, we propose several potential reforms for consideration by neurosurgery residency programs. These proposals aim to introduce transparent, objective metrics and incentivize students to build intentional, high-quality research portfolios. Such changes could benefit disadvantaged applicants—including those without home programs, international graduates, and latecomers—by shifting emphasis from volume to rigor and impact. Implementation will require shared commitment: program directors may welcome reduced review burden and clearer applicant signals, while accountability could be reinforced through consensus statements and pilot programs from neurosurgical organizations. Fundamentally, educational leaders in neurosurgery should collaborate to better define the actual relationship between pre-residency scholarly output and residency success. As neurosurgery is a global specialty, these conversations should always occur in an international context.

1. Adopt a standardized, quality-weighted research scoring system:
  - Frameworks that assess research based on quality—considering publication type, authorship, and impact—can promote meaningful scholarship and discourage superficial publishing. The Arms Race Control Score (ARCS) is 1 prototype currently being piloted through the Stop the Arms Race study, which is assessing its feasibility in residency selection.^40^ While ARCS has significant support, critics have noted limited granularity and the potential to narrow student focus to “what scores well.” For example, ARCS assigns equal weight to basic science and clinical papers, despite longer timelines and greater demands of basic science. This and other critiques should be used to refine ARCS over time. ARCS is designed to complement—not replace—program director judgment, and should be viewed as an evolving, specialty-specific tool that can be improved with feedback from early adopters. A public ARCS calculator already exists and could be integrated into application systems to increase transparency.
2. Introduce explicit caps on research items listed in residency applications:
  - Capping the number of research items applicants can report—such as limiting it to 3 or 5—could help disrupt the zero-sum dynamics fueling the publication arms race.^1^ A hard cap could shift incentives, rewarding applicants not for volume, but for thoughtfully presenting their most meaningful work. However, caps must be implemented carefully, as time and effort vary across disciplines (e.g., basic science vs clinical research). There is a concern that applicants will seek to “end around” the official application process if caps are instituted. Direct applicant contact to programs outside the electronic residency application service application is already rampant, with applicants emailing programs additional letters of recommendation, academic updates, and solicitations for interviews. It should be noted that caps on scholarly output on the application could lead to an increase in the unregulated dissemination of full curriculum vitae by applicants, and this could worsen the inequities of the current system further.
3. Ask applicants to explicitly define their scholarly trajectory or niche:
  - Residency applications could include structured prompts asking applicants to articulate the focus and purpose of their research endeavors. Questions such as, “What scholarly theme or question are you committed to exploring?” or “How do you plan to incorporate research into your clinical practice?” would encourage applicants to think intentionally about the direction of their academic efforts. By shifting the emphasis from quantity to coherence, this approach promotes deeper engagement with a specific area of interest, rather than scattered or opportunistic publication strategies.
4. Recognize impactful nontraditional scholarly activities:
  - Valuing contributions such as quality improvement projects, open-source software development, patents, or medical device innovation would broaden the definition of scholarship and encourage innovation and collaboration. These efforts often demand creativity, technical skill, and sustained commitment, with the potential to improve patient care and healthcare systems. Such activities could be formally incorporated into a revised quality-weighted research score.
5. Pilot objective surgical skill assessments:
  - Incorporating objective assessments of surgical skills into the application process would provide programs a clinically relevant measure of aptitude directly tied to neurosurgical practice. A Mayo Clinic study found that applicants scoring higher on a simulated surgical skills multiple-mini-interview had higher operative competency ratings at the end of intern year.^41^ From an incentive design perspective, incorporating skill-based assessments could shift the focus toward competencies that more accurately predict success in surgical training. However, implementation requires caution: access to surgical training and mentorship varies widely, raising equity concerns. Standardized tasks also risk incentivizing applicants to “teach to the test” by practicing exercises of limited clinical value. Any assessment would therefore need to be carefully designed to ensure fairness and preserve predictive validity.
6. Incorporate standardized subinternship milestone assessments:
  - Residency programs could adopt a uniform, milestone-based tool to assess subintern performance, as proposed by Bowden et al.^42^ This evaluation spans clinical reasoning, procedural skill, professionalism, and communication over a one-month rotation, yielding a cumulative milestone score. The cumulative milestone score distinguishes performance both within and across institutions and offers programs a validated, structured metric to complement traditional criteria—supporting a more holistic and equitable selection process.
7. Conduct continuous longitudinal research examining the predictive value of pre-residency publications on residency and postresidency performance:
  - The neurosurgical community could commit to ongoing, longitudinal studies assessing whether pre-residency research productivity meaningfully predicts success during and after residency. These studies should evaluate a range of outcomes—including clinical performance, academic productivity, board passage rates, faculty evaluations, and long-term career trajectories.
8. Encourage collective guidelines among neurosurgery program directors:
  - Neurosurgery program directors could adopt shared guidelines that explicitly shift the focus from raw publication count to research quality and diverse forms of scholarship. Modeled after consensus statements in other domains of medicine, these principles could discourage curriculum vitae inflation and promote more meaningful research—while allowing customization to align with individual program missions.^43^

## CONCLUSION

The current residency selection landscape has created a publication arms race that prioritizes quantity over quality and burdens students with misaligned incentives. Publication volume has emerged as a proxy for applicant potential—despite its unproven reliability as a measure of clinical or academic excellence.

Neurosurgery is well-positioned to lead meaningful change. By rethinking how research contributions are evaluated and adopting reforms, the field can more accurately recognize genuine academic promise and reduce the pressure to generate low-impact work. Emphasizing quality and coherence in scholarly output will strengthen the academic fabric of neurosurgery and improve equity in access to the field.
